# Androgen Mediated Regulation of Endoplasmic Reticulum-Associated Degradation and its Effects on Prostate Cancer

**DOI:** 10.1038/srep40719

**Published:** 2017-01-16

**Authors:** Yalcin Erzurumlu, Petek Ballar

**Affiliations:** 1 Ege University, Faculty of Pharmacy, Biochemistry Department, Izmir, 35100 Turkey

## Abstract

The endoplasmic reticulum (ER) comprises thirty percent of the newly translated proteins in eukaryotic cells. The quality control mechanism within the ER distinguishes between properly and improperly folded proteins and ensures that unwanted proteins are retained in the ER and subsequently degraded through ER-associated degradation (ERAD). Besides cleaning of misfolded proteins ERAD is also important for physiological processes by regulating the abundance of normal proteins of the ER. Thus it is important to unreveal the regulation patterns of ERAD. Here, we describe that ERAD pathway is regulated by androgen, where its inhibitor SVIP was downregulated, all other ERAD genes were upregulated. Consistently, androgen treatment increased the degradation rate of ERAD substrates. Using several independent techniques, we showed that this regulation is through androgen receptor transactivation. ERAD genes found to be upregulated in prostate cancer tissues and silencing expression of Hrd1, SVIP, and gp78 reduced the *in vitro* migration and malignant transformation of LNCaP cells. Our data suggests that expression levels of ERAD components are regulated by androgens, that promotes ERAD proteolytic activity, which is positively related with prostate tumorigenesis.

Prostate cancer is the second leading cause of cancer mortality and the most prevalent cancer among males with an estimation of more than 3.3 million men in the United States^1^,^2^. Androgen and the androgen receptor (AR), which is a transcription factor of the nuclear steroid receptor family, play a critical role in any stage of normal or neoplastic growth of the prostate. After androgen binding, AR dissociates from heat shock proteins and forms a homodimer. Dimerized AR then acts as a ligand-dependent transcription factor and binds to the androgen response elements (AREs) of androgen-regulated target genes. As a transcription factor, androgen-bound AR recruits RNA polymerase II and a basal transcriptional complex for the transcription of AR target genes^3^. Since androgen target genes are the mediators of several diverse metabolic processes^4^, it is crucial to specifically identify these androgen-responsive genes. Besides normal prostate growth and pathologies, androgen signaling is also critical for female physiology and other male characteristics, such as muscle mass, strength, bone mineral density and neuronal remodeling^5^. There are several diseases that have been associated with androgen signaling besides prostate cancer such as breast cancer, diabetes, metabolic syndrome, cardiovascular diseases and Alzheimer’s disease^5^,^6^,^7^. Therefore, it is important to delineate the biochemical processes that are altered by androgen action.

In addition to their regulation by hormones, prostate cancer cells are also known to be highly secretory. The Endoplasmic Reticulum (ER) is the organelle responsible for the synthesis and maturation of proteins that are destined for the secretory pathways. There is a sophisticated protein quality control mechanism called the ER-associated degradation (ERAD) that eliminates misfolded or unassembled polypeptides and ensures that only fully maturated proteins reach their sites of function. ERAD is also essential for physiological processes by regulating the abundance of normal proteins of the ER, such as monooxygenase cytochrome p450; cholesterol metabolism regulatory proteins 3-hydroxy-3-methylglutaryl-CoA reductase, insulin-induced gene-1 and apolipoprotein B; neurodegenerative disease proteins superoxide dismutase-1 and ataxin-3; and the metastasis suppressor KAI1/CD82^8^,^9^,^10^,^11^,^12^. Considering its critical role on the regulation of cellular homeostasis, it is not surprising that aberrant ERAD is involved in the pathogenesis of many diseases, such as cancer, cystic fibrosis, neurodegenerative diseases, and diabetes^13^.

Understanding the regulation of ERAD is one of the main questions of cellular proteostasis. Some of ERAD factors, namely Hrd1, Hrd3 and Derl1 are reported to be induced upon activation of unfolded protein response (UPR) in yeast^14^,^15^. Ubiquitination of ERAD components also regulates ERAD. For example, autoubiquitination of Hrd1p is required for retrotranslocation in yeast^16^. For mechanism still not clear, deubiquitination enzymes (DUBs) can also act as positive regulators in ERAD^17^. There are two additional specific regulatory patterns for gp78-mediated ERAD. The first mechanism is to control the level of gp78 by Hrd1, which targets gp78 for ubiquitination and proteasomal degradation^18^,^19^. The second mechanism is via the endogenous ERAD inhibitor, namely SVIP, which inhibits gp78-mediated ERAD by competing with p97/VCP and Derlin1^20^.

There is very limited information on ERAD and androgen signaling pathways in prostate cancer cells to date. In 2009, Romanuik *et al*. identified SVIP as one of the novel androgen-responsive genes by sequencing of LongSAGE libraries^21^. Since the previously characterized ERAD inhibitor SVIP found to be negatively regulated by androgen treatment in LNCaP cells, we were prompted to test regulation of ERAD pathway by androgen.

In this study, we showed that ERAD is an androgen-regulated process where both the mRNA and protein levels of ERAD components are regulated with the treatment of the synthetic androgen, R1881. We found that while the level of SVIP, the endogenous ERAD inhibitor, is decreased, all other tested ERAD proteins are increased by the R1881 treatment. This pattern is present in androgen sensitive prostate cancer cells, namely LNCaP and 22RV1, but not in androgen insensitive prostate cancer cells, PC3 and DU145. In addition, we showed that anti-androgen bicalutamide efficiently antagonizes the androgenic induction of ERAD proteins in these cells. Moreover, by using a chemical IRE1α inhibitor we found that regulation of ERAD by androgen is partially or fully independent of UPR. Consistent with androgen-mediated regulation of ERAD genes, R1881 treatment increases ERAD proteolytic activity since the degradation rate of two ERAD substrates, CD3δ and KAI1. Finally, the effect of Hrd1, gp78, and SVIP was evaluated on the cell proliferation rate, wound healing, migration and malignant transformation of LNCaP cells using the *RNAi* approach, and our data suggests that ERAD may be involved in *in vitro* migration and malignant transformation in LNCaP cells.

## Results

### Differential expression of ERAD proteins in prostate cancer cell lines

**Figure 1:**
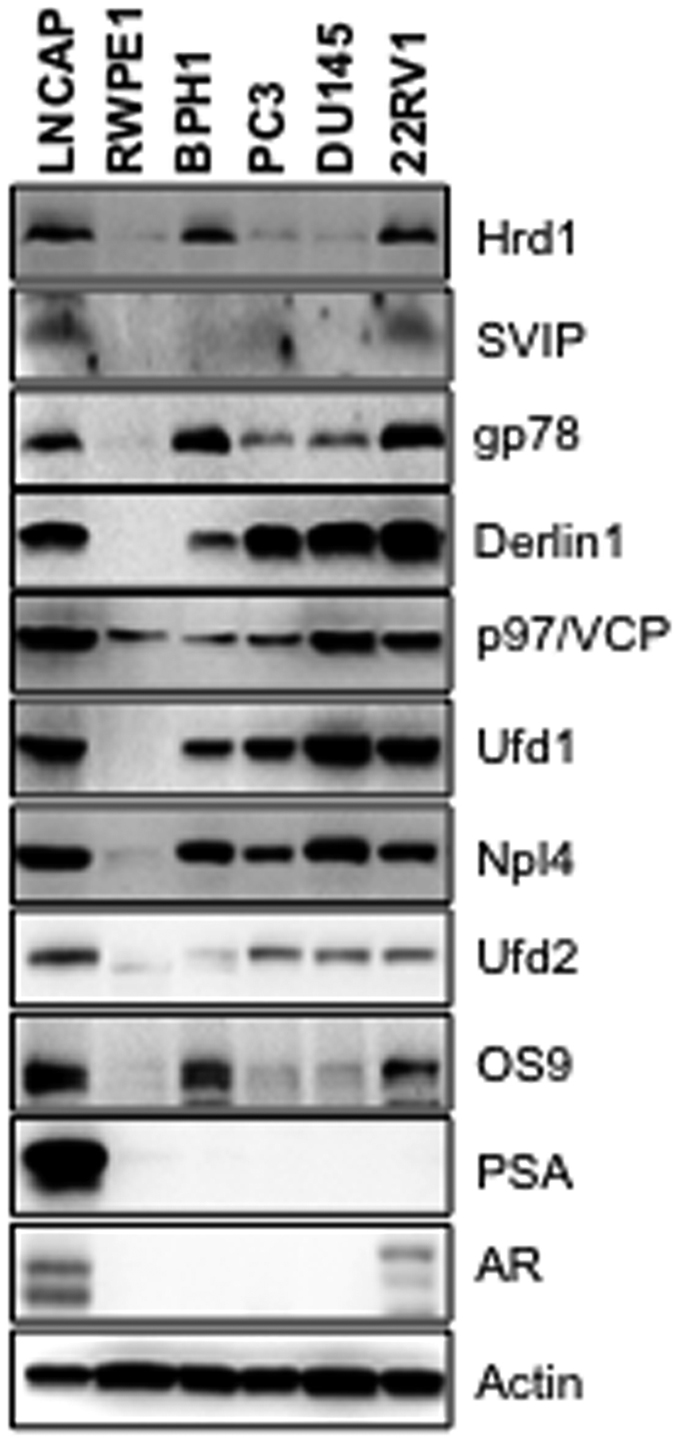
Expression of ERAD components in different prostate cell lines. The expression levels of ERAD components, AR and PSA levels in prostate cell lines were determined by immunoblotting. Actin was used as the loading control in all immunblotting analyses in this study.

To determine the role of ERAD components in prostate tumorigenesis, we first examined their protein expression levels by immunoblotting (IB) in 6 prostate epithelial cell lines. For this aim, two non-tumorigenic prostate cell lines: normal prostate epithelial cell line (RWPE1) and benign prostatic hyperplasia epithelial cell line (BPH1) were utilized as controls. As tumorigenic cell lines, two androgen-sensitive prostate cancer cell lines (LNCaP and 22RV1) and two androgen-insensitive prostate cancer cell lines (DU145 and PC3) were included. Among all the tested ERAD components, two ubiquitin ligases, Hrd1 and gp78, and glycan binding lectin, OS9, were expressed significantly higher in the hyperplastic (BPH1) and androgen sensitive cells (LNCaP and 22RV1); whereas the ERAD inhibitor SVIP was expressed only in the LNCaP and 22RV1 cells (Fig. 1). Almost all of the tested ERAD components except p97/VCP were either not expressed or expressed in very low levels in the normal prostate epithelial cell line, RWPE1 (Fig. 1). In summary, our data indicates that ERAD component levels are all high in androgen-sensitive LNCaP and 22RV1 cells.

### Regulation of ERAD components by androgen

**Figure 2:**
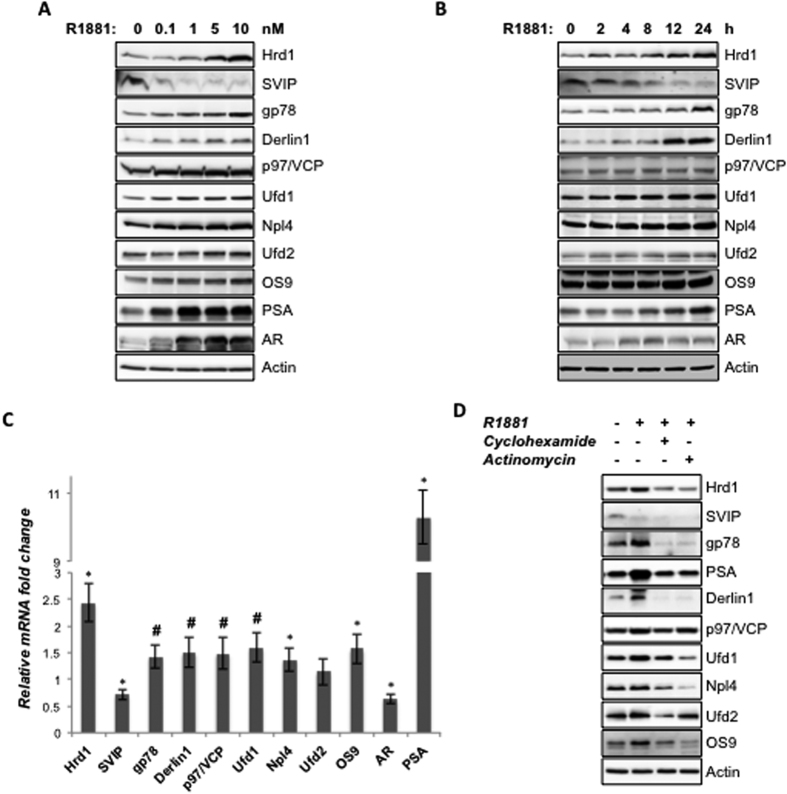
ERAD is regulated by R1881 in a dose- and time-dependent manner. (**A**) Androgen-starved LNCaP cells were treated with R1881 at indicated doses for 24 hour and the levels of ERAD components; AR and PSA were analyzed by immunoblotting using antibodies raised against them. **(B)** Androgen-starved LNCaP cells were treated with 10 nM R1881 for indicated times and expression levels were analyzed as in 2A. **(C)** Androgen-starved LNCaP cells were treated with 10 nM R1881 and the mRNA levels of the indicated genes were investigated using quantitative PCR (QPCR). Controls were treated with vehicle and set to 1. Data represent the mean of two independent biological replicates in triplicates and error bars represent SE. p-values were calculated with respect to vehicle-treated cells by two-tailed equal variance Student’s t-test. (*p < 0.005, ^#^p < 0.05) **(D)** Androgen-starved LNCaP cells were pretreated first with 1 μg/ml cycloheximide or actinomycin for 1 h followed by R1881. 24 h after R1881 treatment, cells were lysed and the expression levels of the proteins of interest were tested using immunoblotting as in 2A.

Since the prostate cancer cell lines with intact androgen receptor showed higher expression levels of all tested ERAD component proteins and ERAD inhibitor of SVIP was reported as one of the novel androgen-responsive genes by sequencing of LongSAGE libraries^21^, we hypothesized that the ERAD pathway might be regulated by androgen. LNCaP cells were treated with increasing concentrations of R1881 (0.1–10 nM) for 24 h and processed for protein expression analyses. R1881 treatment caused a dose dependent increase of E3 ubiquitin ligases, Hrd1 and gp78; retrotranslocation complex members p97/VCP, Ufd1, Npl4 and Derlin1; E3/E4 ubiquitin ligase Ufd2a and glycan binding lectin, OS9. The increase of the expression level of these proteins was in parallel with the dose dependent induction of AR and the endogenous AR target, PSA (Fig. 2A). Interestingly, the level of the ERAD inhibitor SVIP was decreased dose dependently, while the levels of all other ERAD components were increased. Together, this data suggests that the expression of ERAD components is regulated by androgen in a dose-dependent manner, in other words androgen treatment downregulates ERAD inhibitor SVIP levels but upregulates other ERAD genes.

A time-course study was performed in LNCaP cells by using different treatment lengths (2–24 h) with 10 nM R1881. Once again the expression levels of almost all the ERAD components showed significant increase in a time-dependent manner, whereas only ERAD inhibitor SVIP level was decreased (Fig. 2B). Together, our data indicate that ERAD is regulated by *in vitro* androgenic stimulation in a time- and dose-dependent manner.

In order to see whether the effect of androgen on ERAD components is on the protein or mRNA level, we treated LNCaP cells with 10 nM R1881 for 24 h and ERAD genes were tested for their altered mRNA expression levels using RT-qPCR. All the ERAD genes except Ufd2a, showed statistically significant alterations (p < 0.05 for gp78, SVIP, p97/VCP, Ufd1 and p < 0.005 for Hrd1, Derlin1, Npl4 and OS9) on mRNA levels (Fig. 2C). Consistent with protein expression results, R1881 treatment decreased the SVIP mRNA level, whereas increased the mRNA expression of other ERAD genes. In this assay system, PSA was used as a positive control and its mRNA expression was increased 10-fold with 10 nM R1881 treatment (Fig. 2C). Interestingly, our data showed that mRNA level of AR was decreased by R1881 treatment (Fig. 2C), while its protein level was augmented (Fig. 2A). In fact, our results are consistent with a previous publication by Yeap *et al*.^22^, which reports that androgen downregulates AR mRNA transcription, while increases the AR protein expression due to the stabilization of the ligand receptor complex after ligand binding^22^. To summarize, our data suggests that androgen regulates ERAD component levels in both the protein and mRNA level (Fig. 2C). To further analyze whether the androgen-mediated regulation of ERAD is at the gene transcriptional or translational level we pretreated LNCaP cells either with the RNA synthesis inhibitor actinomycin D (1 μg/ml) or the protein synthesis inhibitor cycloheximide (1 μg/ml) and then added R1881 to the medium^23^. Both of these inhibitors significantly abolished R1881 induced expression of Hrd1, gp78, Derlin1, p97/VCP, Npl4, Ufd2 and OS9, where well known AR-target PSA was used as the positive control (Fig. 2D).

**Figure 3:**
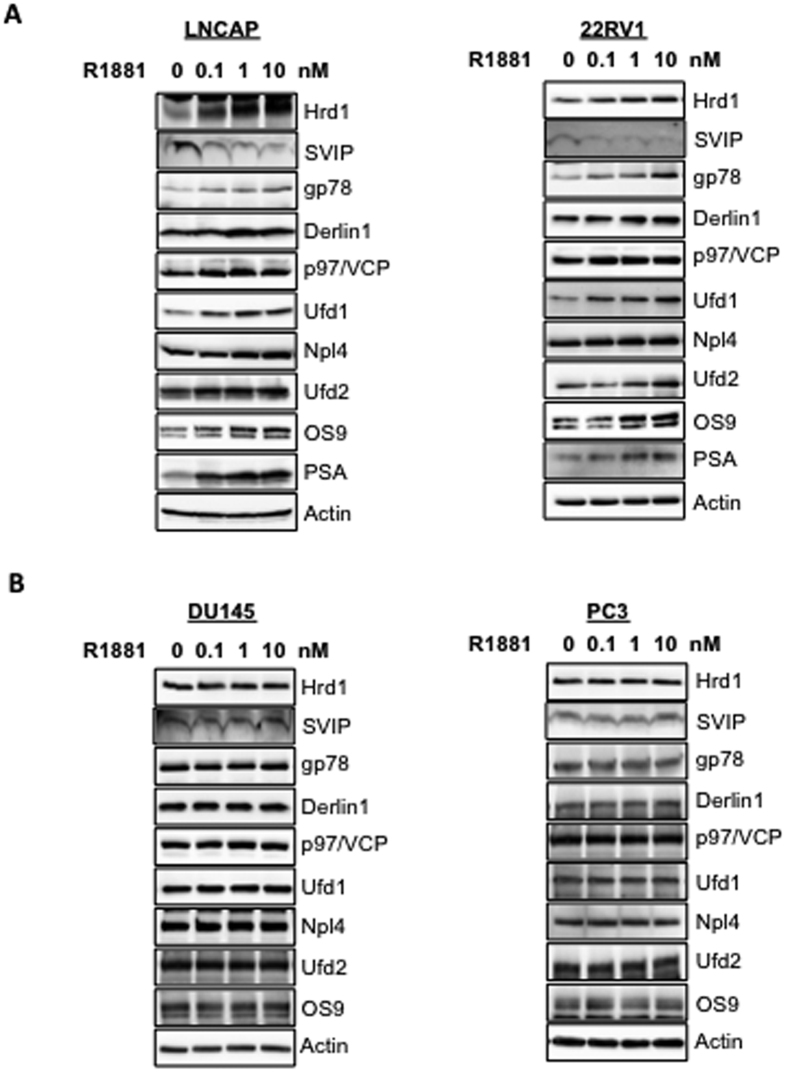
Regulation of ERAD by androgen is present in androgen-sensitive cell lines but not in androgen-insensitive cells. **(A)** Androgen sensitive cells **(B)** Androgen-insensitive cells were treated with R1881 at indicated doses for 24 hour and the level of ERAD components and PSA were analyzed by immunoblotting as in Fig. 2.

In order to see whether the regulation of ERAD by androgen is only limited to metastasis derived androgen-sensitive LNCaP cells, we performed a similar dose-response study with the non-metastasis derived androgen-sensitive 22RV1 cell line and found that all ERAD components were similarly regulated by androgen in both LNCaP and 22RV1 cells (Fig. 3A). This data suggests that regulation of ERAD by androgen is not limited to LNCaP cells since SVIP levels were downregulated, while all other ERAD component expressions were upregulated both in LNCaP and 22RV1. Consistently, ERAD was not regulated by androgen in two AR-negative prostate cancer cell lines, PC3 and DU145 (Fig. 3B).

**Figure 4:**
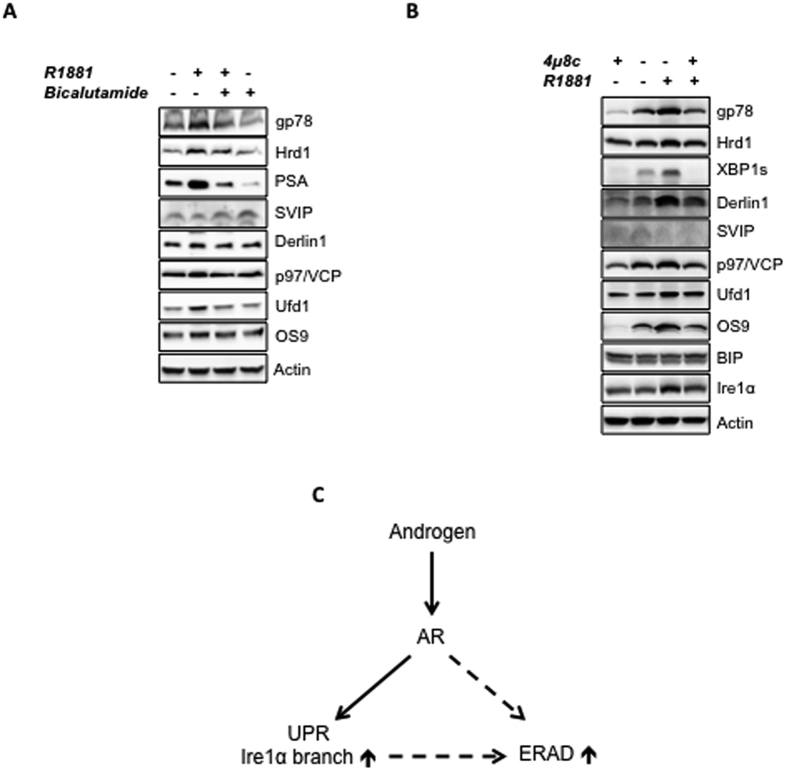
Effect of androgen antagonist and inhibition of IRE1α branch of UPR on the androgen regulation of ERAD. Androgen-starved LNCaP cells were pretreated first with **(A)** 10 μM bicalutamide **(B)** 1 μM 4μ8c for 1 h and then with R1881. 24 h after R1881 treatment, cells were lysed and the expression levels of the proteins of interest were tested using immunoblotting as in 2A. **(C)** Schematic representation of androgen mediated regulation mechanism of ERAD.

Androgens show their biological effects via the intracellular AR, which is a ligand-activated transcription factor and pretreating cells with androgen antagonists abolishes the androgen action. Among several antiandrogen agents, bicalutamide (Casodex) is known to act as a pure androgen receptor antagonist in LNCaP cells^23^. To examine whether androgen mediated regulation of ERAD is mediated via AR transactivation, we pretreated LNCaP cells with the effective dose of bicalutamide (10 μM) for 1 h that was followed by the R1881 treatment. PSA has been used a positive control. Pretreatment of bicalutamide ablated R1881 induced induction of ERAD proteins, such as Hrd1, gp78, Derlin1, p97/VCP, Ufd1, Npl4, Ufd2, OS9. When bicalutamide was present alone in culture, SVIP expression was upregulated, while the downregulation of SVIP level following R1881 treatment was significantly blocked in the presence of bicalutamide. This data suggests that the regulation of ERAD pathway by androgen is specifically mediated via the AR (Fig. 4A).

The levels of Hrd1, Hrd3 and Derlin1 are reported to be enhanced upon activation of UPR in yeast^14^ and a recently published paper described androgen-mediated induction of IRE1α branch and inhibition of PERK signaling of UPR^24^. Thus we wanted to test whether IRE1α induction is responsible for androgen-mediated induction of ERAD by utilizing a chemical IRE1α inhibitor, 4μ8c. R1881 mediated upregulation of ERAD genes such as gp78 was observed both in 4μ8c-treated cells (Fig. 4B, lane 1 versus 4) and in the cells with intact IRE1α pathway (Fig. 4B, lane 2 versus 3). In this assay system we also checked the success of IRE1α inhibition by detecting the expression level of XBP1s, the downstream effector of IRE1α branch. While R1881 treatment caused an upregulation (Fig. 4B, lane 2 versus 3), 4μ8c treatment caused a downregulation on XBP1s expression level (Fig. 4B, lane 1 versus 2). Furthermore, co-treatment of R1881 with 4μ8c did not increase the XBP1s expression compared to the cells treated with only 4μ8c (Fig. 4B, lane 1 versus 4), confirming that unlike ERAD genes, the upregulation of XBP1s expression by R1881 is solely dependent on IRE1α activity (Fig. 4B, lane 2 versus 3). Interestingly, 4μ8c treatment caused significant decrease on the basal expression levels on ERAD component levels (Fig. 4B, lane 1 versus 2), but not on BIP and Ire1α levels. Together our results suggest that regulation of ERAD by R1881 is mediated via AR and is partially or fully independent on the androgen-mediated induction of IRE1α branch of UPR (Fig. 4C).

### Androgen treatment increases ERAD activity

**Figure 5:**
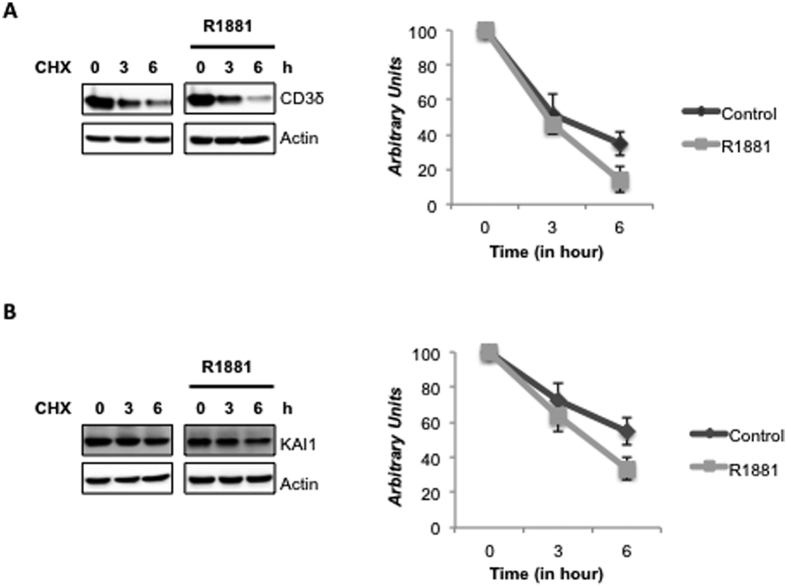
ERAD activity is enhanced by R1881. LNCaP cells were transfected with **(A)** CD3δ and **(B)** KAI1 on 100 mm dishes. Six hours later, cells were splitted onto 6 well dishes. Next day, cells were first androgen-starved and then treated with R1881. Cycloheximide was added into indicated samples 18 h and 21 h after R1881 treatment and cells were harvested 24 hours after R1881 addition. The level of CD3δ and KAI1 was detected by immunoblotting against their tags HA and myc, respectively and quantified by normalizing samples to actin levels. The degradation rates of substrates (*right*) were analyzed using three independent experiments.

In this study we showed that androgen treatment decreases the expression level of the ERAD inhibitor SVIP, while increases the expression levels of all other tested ERAD components; indicating a general induction of the ERAD pathway. Thus, we hypothesized that AR signaling produces homeostatic adjustments in the ERAD activity. To test this hypothesis, we transfected LNCaP cells with a well-known ERAD substrate, CD3δ^25^, and determined its degradation rate using cyclohexamide chase analysis. As seen in Fig. 5A, the degradation rate of CD3δ increased significantly with R1881 treatment in LNCaP cells. We also checked the degradation rate of another ERAD substrate, a transmembrane metastasis suppressor, KAI1^11^. Since the endogenous KAI1 in LNCaP cells was not detectable, we also overexpressed KAI1 by using a plasmid coding for the KAI1 gene for ectopic expression^26^,^27^. As for CD3δ, the degradation rate of KAI1 significantly increased with R1881 treatment (Fig. 5B). Our data strongly suggests that androgen treatment downregulates ERAD inhibitor SVIP and upregulates all other ERAD genes, which in turn enhances ERAD proteolytic activity.

### ERAD components are upregulated in prostate cancer tissues and induce prostate cancer cell proliferation and oncogenicity

**Figure 6:**
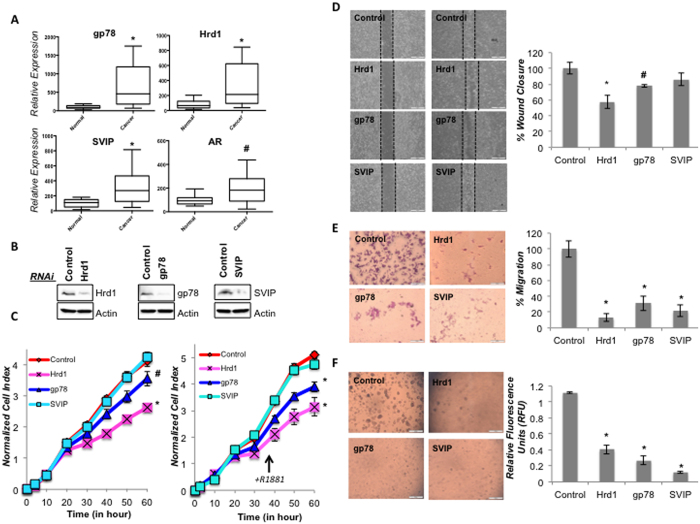
The role of gp78, Hrd1 and SVIP in prostate cancer tumorigenesis. **(A)** Relative expression of some ERAD genes and AR in prostate cancer tissues (n = 39) compared to normal prostate tissue (n = 9) detected by RT-qPCR. Experiment was performed in 3 technical replicates. B-actin gene is used as reference gene. p-values were calculated with respect to vehicle-treated cells by two-tailed equal variance Student’s t-test (*p < 0.005, ^#^p < 0.05). **(B)** LNCaP cells were transfected with siRNA for the indicated genes. Silencing the expression of indicated proteins were analyzed with immunoblotting as in Fig. 2. (**C**) The proliferation rates of cells silenced as indicated were determined with real time cell growth assay of three biological and six technical replicates. Cells were treated with vehicle and R1881 in left and right, respectively. **(D)** Wound healing assay was performed using LNCaP cells seeded on 35 mm dishes with high culture-insert coating (IBIDI). The closure of the gap created by the removal of insert was monitored for three days. Representative images are shown. The analysis of wound closure% was determined using the ImageJ software. Two independent biological and three technical repeats per experiment were used. p-values were calculated with respect to control siRNA transfected cells by two-tailed equal variance Student’s t-test (*p < 0.005, ^#^p < 0.05). **(E)** Boyden chamber assay was done using 24-well transwell chamber as explained in the Material and Methods section. The migrated LNCaP cells on the lower surface of the membrane were fixed and stained with Giemsa. Representative images are shown. Migration was quantified by counting stained cells. The mean percentage of migrated cells compared to control groups were given using the data obtained from two independent biological replicates in triplicates (*p < 0.005). **(F)** The colonogenic assay of LNCaP cells was performed as explained in Material and Methods. Representative microimages are shown. Quantification of colony formation of cells was performed with CyQuant GR dye using a fluorometer (*p < 0.005).

We checked the expression level of some ERAD genes in prostate tissue samples by using a Prostate Cancer Tissue Array containing 9 normal and 39 prostate cancer tissues. Our data revealed that gp78, Hrd1, SVIP and AR showed increased expression (p < 0.05 for AR and p < 0.005 for gp78, Hrd1, SVIP) in prostate cancer tissues (Fig. 6A). As expected prostate cancer patient samples had diverse levels of ERAD component mRNA detected by RT-qPCR. It is noteworthy to mention 51% of prostate tumors (20 of 39) and 25% of tumors (10 of 39) had 5 fold higher gp78 mRNA expression and Hrd1 mRNA expression, respectively, compared to normal prostate tissue controls (n = 9) (Table 1).

To further investigate the potential role of ERAD in prostate tumorigenesis, we transiently silenced Hrd1, gp78 or SVIP expression in LNCaP cells (Fig. 6B) and analyzed the cell proliferation rates of cells by measuring the impedance-based signals every 30 min for 60 h using a real time cell analyzer system. Our data showed that silencing Hrd1 or gp78 expression caused significant reduction (p < 0.005 for Hrd1, p < 0.05 for gp78) in cell growth rate of LNCaP cells (Fig. 6C, left). We obtained similar results when LNCaP cells were treated with 10 nM R1881, where cells with silenced Hrd1 or gp78 expression had slower proliferation rate (p < 0.005 for Hrd1, gp78). On the other hand, there was no change detected in the proliferation rate of SVIP silenced cells. In order to test the role of ERAD components on the motility of LNCaP cells, an *in vitro* wound healing model was carried out using IBIDI linear exclusion systems which prevents cell growth in a predefined, standardized region. After removal of the insert, cells were monitored for their motility. Our data indicates that silencing of gp78 and Hrd1 resulted in a decrease in the rate of wound closure (p < 0.005 for Hrd1, p < 0.05 for gp78) (Fig. 6D).

Next, we analyzed the effect of selected ERAD genes on the migration of LNCaP cells using the Boyden Chamber assay to assess their serum-stimulated chemokinesis. Our data indicates that silencing Hrd1, gp78 or SVIP expression decreased the serum stimulated-migration ability of LNCaP cells (Fig. 6E). Lastly, soft agar assay was performed to examine the effect of Hrd1, gp78 or SVIP on anchorage independent growth, which is a hallmark of malignant transformation. Instead of using the classical soft agar assay, which involves manual counting of colonies, we used a 96-well fluorescence cell transformation assay. This assay system also has a relatively shorter incubation time (around 6 days), which makes it possible to work with cells that are transiently transfected with siRNAs. As seen in Fig. 6F, silencing of Hrd1, gp78 or SVIP caused a significant decrease on both the size and number of colonies, as well as a decrease in the measured fluorescence intensity (Fig. 6F). In conclusion, our data suggests that silencing of Hrd1 and gp78 affect the proliferation rate, whereas Hrd1, gp78 and SVIP have role in malignant transformation of prostate cancer cells.

## Discussion

ERAD is the most effective, rapid and direct means to remove misfolded proteins. Besides degrading these potentially toxic misfolded proteins, ERAD also regulates the level of some properly folded proteins, such as HMG-CoA reductase; rate-limiting enzyme of cholesterol biosynthesis; apolipoprotein B, assembly factor of cholesterol-containing liposomes, and KAI1, tumor metastasis suppressor^8^,^9^,^11^. Considering its critical role in the regulation of cellular homeostasis, it is believed that any aberration on ERAD has significant effects on cell physiology. To date, there are almost 70 ERAD substrates linked to a variety of human diseases including cancer, neurodegenerative diseases and diabetes^13^. Therefore, there is an ongoing extensive research on the ERAD substrates, their association with diseases, elucidation of the steps of ERAD mechanism and its regulation. Despite all of these studies, little is known about the intracellular regulation of mammalian ERAD. This study, to the best of our knowledge, is the first study to characterize the androgen-mediated regulation of the ERAD pathway.

As a multistep process, more than 50 proteins are involved in ERAD^28^,^29^. In this study, we examined ERAD through several selected genes including OS9, which functions in substrate recognition and targeting; gp78 and Hrd1, E3 ubiquitin ligases; Ufd2a functioning as E3/E4 ubiquitin ligase; Derlin1, p97/VCP, Ufd1 and Npl4 as components of the retrotranslocation complex and SVIP, which is the first identified endogen ERAD inhibitor.

In 2009, SVIP was reported as one of the novel androgen-responsive genes by sequencing of LongSAGE libraries^21^. Considering that SVIP is an ERAD inhibitor and found to be negatively regulated by R1881 treatment in LNCaP cells^21^, we tested the regulation of the ERAD pathway using LNCaP prostate cancer cell line using the synthetic androgen, R1881. Besides the proliferation pattern of LNCaP, its expression in differentiated secretory pathway and the control of some cellular pathways, such as lipid synthesis and accumulation also remains to be androgen responsive^30^,^31^.

In this study, we observed that the expression levels of ERAD components are highest in androgen-responsive prostate cancer cells among other tested prostate cells (Fig. 1). The expression of some ERAD components was also present in androgen insensitive prostate cancer cells and hyperplastic prostate cells. However, almost no ERAD components, except p97/VCP, were detected in normal prostatic epithelial cells (Fig. 1). Our data strongly suggests that all tested ERAD components, except SVIP, were upregulated by R1881 treatment in a dose- and time-dependent manner (Fig. 2). However, SVIP, the endogen inhibitor of ERAD, was downregulated by androgen treatment (Fig. 2). The regulation of ERAD by androgen is not limited to LNCaP cells, but also to another androgen responsive cell line, 22RV1, which has a similar androgen-mediated ERAD regulation pattern (Fig. 3). The effect of androgen observed on the ERAD components levels was mediated through the AR, since bicalutamide (androgen antagonist) pretreatment reduced the effect of R1881 on ERAD protein levels (Fig. 4). Since the ERAD inhibitor protein SVIP was downregulated while all other tested ERAD components were upregulated, we hypothesized that ERAD activity should be augmented in the R1881-treated LNCaP cells. Indeed, our cycloheximide chase assay results suggest that the ERAD substrates were degraded faster in R1881-treated cells when compared to control cells (Fig. 5).

In a prostate cancer tissue panel of patients we found the mRNA expression levels of Hrd1, gp78, and SVIP are upregulated in prostate cancer (Fig. 6A). Thus, we also examined the role of Hrd1, gp78, and SVIP on prostate tumorigenesis. The silencing expression of ubiquitin ligases, Hrd1 and gp78 (but not SVIP), decreased the proliferation rate of LNCaP cells both with and without R1881 treatment (Fig. 6C). Surprisingly, silencing of all tested ERAD components very drastically inhibited *in vitro* transwell migration and colony formation in LNCaP cells (Fig. 6E and F), suggesting that both positive (Hrd1, gp78) and negative (SVIP) regulators have similar role in malignant transformation of LNCaP cells. This might be due to multiple functions that have been reported for SVIP. Besides being an ERAD inhibitor, SVIP has been characterized as a regulator of autophagy pathway^32^ and as p97/VCP independent myelin protein component in neurons^33^. More extensive work on the mechanisms of tumor invasion and metastasis needs to be performed in the future including *in vivo* tumor growth assays.

While we were working on this manuscript, a report identifying a divergent androgen regulation of UPR in prostate cancer cells was published^24^. In this paper, it was suggested that androgens activate the inositol requiring enzyme 1α (IRE1α) branch, but inhibit the protein kinase RNA-like endoplasmic reticulum kinase (PERK) signaling in prostate cancer cells. In accordance with these findings, we also found that the IRE1α branch was activated and XBP1s expression level were significantly increased by R1881 treatment in LNCaP cells (Fig. 4B). Our data in Fig. 4B indicates gp78, Hrd1, OS9, Derlin1, Ufd1 expression levels were also upregulated by R1881 in cells that IRE1α activity was inhibited by using 4μ8c (Fig. 4B, lane 1 versus 4). Therefore, upregulation of ERAD by R1881 is partially or fully independent of androgen-mediated UPR induction. However, it is interesting that treatment with only 4μ8c caused decrease on the expression of especially gp78, Os9, Ufd1 and Derlin1, which might be the reason that 4μ8c treated cells have lower degree of upregulation by R1881 than the response obtained in cells with intact IRE1α activity (Fig. 4B).

In an effort to find putative binding sites for AR (ARE) we examined the human genomic sequences of ERAD genes tested in this study using *in silico* MatInspector bioinformatic tool (Genomatix Software, Munich, Germany, http://www.genomatix.de). A restrictive threshold of 0.85 and V$GREF matrix were used for prediction of putative AREs^34^. This scanning results in the identification of three putative ARE sites for Hrd1, one for gp78, six for p97/VCP gene, seven for Ufd1, one for SVIP, six for OS-9, four for Ufd2a and six for Npl4 (Supplemental Table 1). Further tests are required to analyze whether those putative AREs are really functioning. This *in silico* screening results together supports our experimental results suggesting that ERAD is regulated with androgen action via androgen receptor.

Besides its effect on prostate, androgens play several roles in different tissues such as androgen-mediated augmentation of the axonal regeneration after peripheral nerve injury^35^. Androgens might also act directly in the AR-containing cell populations in the nerve to enhance axonal growth and myelination^36^. Recently, SVIP was identified as a novel compact myelin protein in the sciatic nerve, independent of its interaction with p97/VCP suggesting another role of SVIP in the central and peripheral nervous systems, in addition to being an ERAD inhibitor^33^. Sciatic nerves from adult male and females rats were previously reported to contain both the AR mRNA and protein. In addition, endoneurial fibroblasts have implications for site of androgen actions and the AR might mediate the effects of androgens in the neuromuscular systems^36^. The expression of Glycoprotein Po (Po) and peripheral myelin protein 22 (PMP22), two proteins that play a crucial role in the structure of peripheral myelin, were shown to be modulated by androgens in the sciatic nerve in adult male rats^37^. Moreover, the age-related reduction of Po and myelin basic protein expression was associated with myelin degeneration, which was partially reversed by steroid derivatives^38^. It is noteworthy to mention that similar to Po and myelin protein 22; SVIP, the novel myelin protein is also found to be regulated by androgen in this study. Therefore, the androgen regulation of ERAD genes might be of great importance in other systems and pathologies besides prostate cancer.

In conclusion, our findings suggest that protein and mRNA expression levels of ERAD components are regulated by androgens, that promotes ERAD proteolytic activity, which is positively related with prostate tumorigenesis.

## Materials and Methods

### Materials

All cell culture grade reagents including media, fetal bovine serum (FBS), and growth factors were obtained from either Life Technologies or LONZA. Polyclonal antibodies against Hrd1 (147773), gp78 (9590), Npl4 (13489), Derlin1 (8897), CHOP (2895), PSA (5365), AR (5153), PERK (3192), IRE1a (3294), XBP1-s (12782) were purchased from Cell Signaling Technology. Mouse monoclonal antibodies against p97/VCP (612182), Ufd1 (611642) and Ufd2a (611966) were obtained from BD Transduction Laboratories. Anti-BIP (G90043), anti-actin (A5316), anti-HA (H9658) and anti-myc (M4439) antibodies were from Sigma Aldrich; anti-KAI1 (sc17752) from SantaCruz; anti-OS-9 (ab19853) from Abcam and HRP-conjugated anti-mouse or anti-rabbit IgG was purchased from Pierce. Polyclonal anti-SVIP antibody was described previously^20^.

Actinomycin D, Tunicamycin, Cycloheximide were purchased from Calbiochem and Bicalutamide from Sigma Aldrich.

### Cell culture and treatments

Human prostate cell lines RWPE-1 (normal prostate epithelial cell), 22RV1 (prostate adenocarcinoma), PC3 (prostate *adenocarcinoma*, bone *metastatic* site), DU145 (prostate *adenocarcinoma*, brain *metastatic* site) and LNCaP (prostate *adenocarcinoma*, lymph node *metastatic* site) were obtained from American Type Culture Collection (ATCC, USA), while BPH-1 (benign prostatic hyperplasia epithelial cell line) was purchased from Deutsche Sammlung von Mikroorganismen und Zellkulturen (DSMZ, Germany). The DU145 and PC3 cell lines were cultured and routinely passaged in DMEM/F12 media containing 10% FBS, while LNCaP and 22RV1 cell lines were propagated in RPMI 1640 containing 10% FBS. RWPE-1 was cultured in Keratinocyte Serum-Free Medium supplemented with 5 ng/ml EGF, 0.05 mg/ml bovine pituitary extract, and 1% Pen-Strep antibiotics cocktail. BPH-1 was propagated in RPMI 1640 containing 20% heat inactivated FBS, 20 ng/ml testosterone, 5 μg/ml transferrin, 5 ng/ml sodium selenite and 5 μg/ml insulin.

All the compounds were prepared as a 1000-fold concentrated stock in the solvent, DMSO or ethanol, thus final concentration of solvent did not exceed 0.1%.

All the hormone treatments and *RNAi* applications were performed in LNCaP cells that are below passage 15.

In order to remove steroids and growth factors during hormone treatment, LNCaP cells were grown in starvation medium containing 2% and 0.5% CT-FBS (Charcoal treated-FBS) for 2 days and 1 day, respectively. Cells were then exposed to R1881 as indicated in each experiment. 22RV1 cells were also treated with hormone following the same protocol used for LNCaP cells.

In indicated experiments, after serum starvation, cells were first pretreated either with 10 μM bicalutamide, 1 μg/ml actinomycin or 1 μg/ml cycloheximide for 1 h and then treated with 10 nM R1881 synthetic androgen.

In the cycloheximide chase assay, cells were treated with either with 10 nM R1881 or ethanol as control. After 18 h and 21 h of R1881 treatment, 25 μg/ml CHX was added into 6 h and 3 h samples, respectively. Samples were harvested 24 hours after the initiation of the R1881 treatment.

Transfections were performed either with Lipofectamin-2000 (Invitrogen) or X-tremeGENE HP (Roche) following instructions of manufacturer.

### Protein preparation and Immunoblotting (IB)

Cell lysates were prepared by homogenizing cultured cells in RIPA buffer (1xPBS, 1% nonidet P-40, 0.5% sodium deoxycholate, and 0.1% SDS, pH 8.0). After removal of insoluble materials by centrifugation at 14.000 rpm for 10 min at 4 °C, protein concentrations were determined using BCA protein assay kit (Thermo Scientific). Typically, 40 μg of total cellular protein were used for immunoblotting. Samples were denatured in 6x Laemmli buffer at 95 °C for 5 min and were separated on either handcast polyacrylamide gels or gradient precast ready gels (BioRAD). Gels were transferred onto PVDF membranes (Millipore). Following classical immunoblotting steps (blocking, incubating with primary and secondary antibodies), proteins were visualized using enhanced chemiluminescence (BioRAD) by Fusion FX7 (Vilber Lourmat). Densitometric analyses of protein bands were performed using ImageJ software (http://imagej.nih.gov/ij/).

### Total RNA isolation and Expression Analysis by Quantitative RT-PCR

The total RNA was isolated using Total RNA Isolation Kit (Norgen) following the manufacturer’s instructions. RNA concentration and purity were determined by Nanovette (Beckman Coulter). cDNAs were synthesized using the ProtoScript II First Strand cDNA Synthesis Kit (NEB) using 1 μg of total RNA and oligo dT primers according to the manufacturer’s instructions. The gene expression analysis, quantitative RT-PCR was performed using The SYBR Green I Mastermix (Roche) and LightCycler480 thermocycler (Roche). Specific primers were designed against ERAD genes, PSA and AR, and all primer sequences are listed in Supplemental Table 2. Twenty-microliter reactions were performed with 300 nM of primer pairs. Fold change for the transcripts were normalized to the housekeeping gene TBP1 (TATA-Box Binding Protein1, general RNA polymerase transcription factor, M5564)^39^. The following PCR conditions were used: denaturation at 95 °C for 10 min, followed by 45 cycles of: 10 s at 95 °C, 10 s at 60 °C, and 15 s at 72 °C. For relative quantification, reaction efficiency incorporated ΔΔCq formula was used. Two independent biological replicates with three technical replicates per experiment were used for each PCR. For patient samples, Origene TissueScan Prostate Cancer Tissue Array III (HPRT503) containing 46 tissues covering 39 prostate cancer tissues (18 Stage 2, 19 stage3, 2 Stage4) and 9 normal tissues was used in (3 technical replicas).

### siRNAs, Plasmids and Transfection

Silencer® Negative Control siRNA #1, gp78 (siRNA ID: 110862, sense sequence: CGUAUGUCUAUUACACAGA), SVIP (sense sequence: GACAAAAAGAGGCUGCAUC), Hrd1 (siRNA ID: 124188, sense sequence: CCGUUUUUCGGGAUGACUU) were ordered from Ambion^20^,^40^.

pCI-CD3δ-HA has been previously described^41^. pCMV6-KAI1-myc is obtained from Origene.

### Proliferation, colony formation and migration assays

Proliferation rate of LNCaP cells was monitored using real-time cellular analysis system (xCELLigence, ACEA) measuring impedance-based signals. 7500 cells/well were seeded into 96-well E-plate (ACEA) and cell proliferation was monitored every 30 min for 60 h. Data was expressed as “cell index”, which is defined as “impedance of the well with cells” minus “the background impedance”. Normalization was done at 12 hours, where LNCaP cells were attached and regained their morphology. Three independent biological and eight technical repeats per experiment were used.

For the wound healing assay, 35 mm dishes with high culture-insert coating (IBIDI) consisting of two reservoirs was utilized. After confluent monolayers of LNCaP cells were established on dishes, the insert was gently removed creating a gap of 500 μm. The closure of the gap was monitored for three days and images were taken using Olympus CKX41 microscope. The analysis of wound closure % was determined by using the ImageJ software (http://imagej.nih.gov/ij/). Three independent biological and two technical repeats per experiment were used.

Boyden chamber assay was performed to assess the migration rate by using a 24-well transwell chamber that includes a porous polycarbonate membrane with 8-μm pore size (Corning). Serum-starved LNCaP cells (10000 cells in medium with 0.5%CT-FBS) were seeded onto the Transwell filters (upper chamber). To stimulate cell migration through the membranes, 20% FBS was added to the lower chamber as a chemoattractant. The cells were kept at 37 °C in a CO_2_ incubator for 48 hours. The migrated LNCaP cells on the lower surface of the membrane were fixed with methanol and stained with 0.2% crystal violet solution (Sigma Aldrich). Migration was quantified by counting stained cells and the results were expressed as the mean percentage of migrated cells compared to control groups.

Soft agar colony formation assay was performed with CytoSelect cell transformation assay (Cell Biolab, Inc.) following manufacturer’s instructions. Equal volumes of 2xRPMI-1640 with 20% FBS and 1.2% agar solution were mixed and transferred onto wells in a 96-well plate. Cell suspensions prepared in 25 μl were mixed with 25 μl of 2xRPMI-1640 with 20% FBS and 25 μl of 1.2% agar, and then placed on the solidified bottom agar layer. After the addition of 100 μl of 2xRPMI-1640 containing 10% FBS to each well, the plates were incubated for 6 days under conventional cell culture conditions. The medium was changed every 2–3 days. The images of colonies were taken using Olympus CKX40 microscope. Colonies were lysed and quantified with CyQuant GR dye using a fluorometer equipped with a 485/520 nm filter set (Varioscan, Thermo Scientific).

### Statistics

Data are presented as means ± standard deviation (SD). The statistical significance of differences between groups was assessed by by two-tailed equal variance Student’s t-test using GraphPad Prism software. Values of p < 0.05 were considered significant.

## Additional Information

**How to cite this article:** Erzurumlu, Y. and Ballar, P. Androgen Mediated Regulation of Endoplasmic Reticulum-Associated Degradation and its Effects on Prostate Cancer. *Sci. Rep.* **7**, 40719; doi: 10.1038/srep40719 (2017).

**Publisher's note:** Springer Nature remains neutral with regard to jurisdictional claims in published maps and institutional affiliations.

## Supplementary Material

Supplemental Table 2

Supplemental Table 1

## Figures and Tables

**Table 1:** Relative mRNA expression levels of gp78, Hrd1, SVIP and AR in prostate cancer tissues (n = 39) compared to normal prostate tissue controls (n = 9).

	Relative Expression levels		
	≤1 fold	1–5 fold	≥5 fold
gp78	3	16	20
Hrd1	10	19	10
SVIP	9	22	8
AR	11	26	2
