# Supplementary material for: Androgen Mediated Regulation of Endoplasmic Reticulum-Associated Degradation and its Effects on Prostate Cancer
Source: Sci Rep. 2017 Jan 16;7:40719. doi: 10.1038/srep40719 (PMC5238502; doi:10.1038/srep40719)
Supplement: Supplemental Table 2 [file srep40719-s1.doc]

Supplementary Info

**ANDROGEN MEDIATED REGULATION OF ENDOPLASMIC RETICULUM-ASSOCIATED DEGRADATION AND ITS EFFECTS ON PROSTATE CANCER**

Yalcin Erzurumlu^1^ and Petek Ballar^1,§^

^1^ Ege University, Faculty of Pharmacy, Biochemistry Department, Izmir, 35100 Turkey

^§^ To whom correspondence should be addressed: Petek Ballar, Ege University, Faculty of Pharmacy, Department of Biochemistry, 35100, Izmir, TURKEY, Tel.: +90-232-3113141; Fax: +90-232-3885258; Email: [petek.ballar@ege.edu.tr](mailto:petek.ballar@ege.edu.tr)

**Supplemental Table 1:** **Transcription factor binding site analysis of the ERAD gene promoter regions according to the MatInspector.** Androgen receptor binding sites have been highlighted in yellow. (Excel file)

**Supplemental Table 2:** **Primers used in gene expression analysis studies**
